# Localization of Interictal Epileptiform Activity Using Magnetoencephalography with Synthetic Aperture Magnetometry in Patients with a Vagus Nerve Stimulator

**DOI:** 10.3389/fneur.2014.00244

**Published:** 2014-11-27

**Authors:** Jennifer R. Stapleton-Kotloski, Robert J. Kotloski, Jane A. Boggs, Gautam Popli, Cormac A. O’Donovan, Daniel E. Couture, Cassandra Cornell, Dwayne W. Godwin

**Affiliations:** ^1^Department of Neurology, Wake Forest University School of Medicine, Winston-Salem, NC, USA; ^2^Department of Neurology, William S. Middleton Memorial Veterans Hospital, Madison, WI, USA; ^3^Department of Neurology, University of Wisconsin School of Medicine and Public Health, Madison, WI, USA; ^4^Department of Neurosurgery, Wake Forest University School of Medicine, Winston-Salem, NC, USA; ^5^Department of Neurobiology and Anatomy, Wake Forest University School of Medicine, Winston-Salem, NC, USA

**Keywords:** magnetoencephalography, synthetic aperture magnetometry, vagus nerve stimulator, epilepsy, epilepsy surgical evaluation

## Abstract

Magnetoencephalography (MEG) provides useful and non-redundant information in the evaluation of patients with epilepsy, and in particular, during the pre-surgical evaluation of pharmaco-resistant epilepsy. Vagus nerve stimulation (VNS) is a common treatment for pharmaco-resistant epilepsy. However, interpretation of MEG recordings from patients with a VNS is challenging due to the severe magnetic artifacts produced by the VNS. We used synthetic aperture magnetometry (g2) [SAM(g2)], an adaptive beamformer that maps the excessive kurtosis, to map interictal spikes to the coregistered MRI image, despite the presence of contaminating VNS artifact. We present a series of eight patients with a VNS who underwent MEG recording. Localization of interictal epileptiform activity by SAM(g2) is compared to invasive electrophysiologic monitoring and other localizing approaches. While the raw MEG recordings were uninterpretable, analysis of the recordings with SAM(g2) identified foci of peak kurtosis and source signal activity that was unaffected by the VNS artifact. SAM(g2) analysis of MEG recordings in patients with a VNS produces interpretable results and expands the use of MEG for the pre-surgical evaluation of epilepsy.

## Introduction

Magnetoencephalography (MEG) is a neurophysiological technique that non-invasively measures the biomagnetic activity of the brain with high temporal and good spatial resolution [~2–3 mm (1), and possibly better (2–5)]. Because neuromagnetic activity ranges from 10 fT to 10 pT, the superconducting quantum interference device (SQUIDs) used to detect magnetic fields must be extremely sensitive (1, 6, 7). However, the required sensitivity gives rise to a susceptibility to environmental noise and magnetic artifacts of much larger amplitude (8, 9). Sources of artifact distant from the patient can be limited by placing the MEG scanner in a magnetically shielded room and by using reference gradiometry to cancel sources of external environmental noise (10–14). However, this approach is not sufficient to minimize sources of artifact closer to, or positioned within the patient.

One of the most common clinical uses of MEG is to localize the generators of interictal epileptiform activity (e.g., interictal spikes), often as part of a pre-surgical evaluation in individuals with pharmaco-resistant epilepsy (15–20). A frequent non-pharmacologic therapy for pharmaco-resistant epilepsy is vagus nerve stimulation (VNS), in which stimulator leads are implanted around the left vagus nerve in the neck and a titanium case with a generator and battery is placed into the chest. However, in 25–33% of patients, VNS does not provide satisfactory improvements in seizure control (21, 22) and therefore surgical resection may be reconsidered in these patients. Unfortunately, the magnetic fields produced by a VNS, even when turned off, are up to an order of magnitude greater than those produced by brain activity, potentially limiting the use of MEG to identify the seizure onset zone(s) in individuals with a VNS. Some recently developed techniques have been used to remove these artifacts, including signal space separation (SSS) (23) and temporally extended signal space separation (tSSS) (24, 25).

Synthetic aperture magnetometry (SAM) is an adaptive beamformer based on a constrained minimum variance beamformer (13, 26). One variant of this method, synthetic aperture magnetometry (g2) [SAM(g2)], images the excess kurtosis associated with interictal or ictal spikes, simultaneously mapping the putative generators and reconstructing the source signal series from the local maxima associated with these generators (27–34). The reconstructed source signal series can be considered to be a virtual depth electrode in that the source signal series provides a continuous estimate of the neuromagnetic activity arising from the voxel and bears good similarity to the neural activity detected by invasive monitoring (35). SAM has also been used successfully to remove the artifacts associated with a contact heat evoked potential stimulator (10), dental hardware (32, 36), and eye movements (37). Because, it is a spatial filter (13, 26) and has the desired properties of estimating the kurtotic signature of all brain voxels while suppressing extraneous signals, we hypothesized that SAM(g2) would selectively minimize the spatially distinct artifacts associated with a VNS while preserving neural activity.

We report a series of patients with VNS implants who underwent a MEG recording in the course of treatment. In this study, MEG was used to localize interictal epileptiform activity for eight patients with implanted VNS devices. Previous localization attempts utilizing equivalent current dipole (ECD) modeling for these patients had been equivocal, and as SAM has been successfully used to remove spatially distinct artifacts previously (10, 32, 36, 37), we hypothesized that SAM(g2) would be capable of localizing interictal epileptiform activity in these patients while minimizing the large amplitude but spatially distinct magnetic artifact due to the VNS.

## Materials and Methods

### Patients

All patients were identified by a search of electronic medical records at Wake Forest Baptist Medical Center (IRB 15854). Selection criteria simply included any patient with a VNS who had a MEG recording, covering the dates from March 2006 through February 2012. No other attempts were made to limit or exclude subjects, and a total of eight patients (five females) with a VNS implant were scanned during this time frame. Seven of the patients were adults (age range 31–63 years), and the eighth was a child (6 years old). The medical records for the identified patients were checked for scalp EEG, invasive monitoring, structural imaging, and metabolic imaging, and if available, such data were collected as well. Clinical outcomes were determined by reviewing notes from follow-up office visits.

### MEG and EEG acquisition

Each patient’s VNS was turned off at least 2 days prior to the MEG recording to allow for dissipation of magnetic fields produced by activity of the VNS and thereby preventing possible damage to the SQUIDs. Patients were sleep-deprived on the night before the scan and were not required to withhold any medications. The MEG recordings were performed in a magnetically shielded room with a CTF Systems, Inc., 2005 whole-head MEG system containing 275 first-order axial gradiometers and 29 reference magnetometers and gradiometers. Fiducial coils were placed on the nasion and left and right preauricular points for each patient to permit continuous head localization during the recordings (38, 39). Head motion is generally minimal because patients usually sleep during the scans. The average patient movement during these scans was 3.33 ± 0.78 mm (average ± SEM). Simultaneous EEG was recorded with whole-scalp coverage using the International 10–20 system of electrode placement. Both MEG and EEG were sampled at 600 Hz with a bandwidth of 0.05–150 Hz. Data were recorded in 4 min epochs, for a total of 10–12 epochs. The total recording duration for each subject was 40–48 min.

### MEG analysis

All analyses were performed in the CTF MEG™ Software package (MISL, Coquitlam, BC, Canada). MEG data were processed offline and received synthetic third gradient balancing and DC offsetting. The data were filtered from 3 to 70 Hz with a notch filter at 60 Hz. All raw data were reviewed and rare periods of muscle artifact were manually marked and were not used for analysis. Next, SAM(g2) was used to generate statistical parametric brain maps (with 5 mm voxels) of excess kurtosis within the frequency band of 20–70 Hz for each 4 min epoch. The 20–70 Hz frequency band is chosen to isolate the sharp (highly kurtotic) epileptiform activity from ongoing brain rhythms that are smoother. These maps were aligned with each patient’s structural MRI scan on the basis of overlapping common fiducials. The source signal series were reconstructed within the frequency band of 3–70 Hz at all local maxima with an inter-peak spacing >10 mm in each map for g2 ≥ 3 (27–31, 33, 35, 40). Spike times were automatically marked in the source signal series and compared to the activity visible in the simultaneous EEG (32). No other analysis was performed to filter out artifact arising from the VNS implants. For clinical reporting and for the plots presented here, estimates of the irritative zone responsible for the interictal activity were provided by thresholding the SAM(g2) maps according to the value of the full width half maximum of the most kurtotic peak.

## Results

Despite the presence of excessively large amplitude artifacts (>50 pT peak to peak) in the raw data due to the VNS implants, SAM(g2) analysis of the MEG data detected foci of high kurtosis in all eight patients and successfully minimized the VNS-induced artifact in the source signal series data for all patients. Epileptiform activity in the same region was compared to other localizing studies for each patient. Epileptiform activity was found on the simultaneously recorded EEG of five of eight patients. Structural abnormalities were identified in four cases and MEG foci clustered over these abnormalities in two of four cases. Additionally, four of the patients underwent invasive monitoring following their MEG recordings. A detailed description of each patient follows.

### Patients

#### Patient 1

A 31-year-old right-handed woman with implantation of a VNS 3 years prior to her MEG recording presented for further evaluation. Since age 5 years old the patient had focal seizures that began with a “funny feeling,” flushing, and head turning to the right. Some seizures terminated at this point, while at other times her seizures progressed to impair awareness and/or evolved into a bilateral convulsive seizure. Treatment with acetazolamide, carbamazepine, valproic acid, and levetiracetam failed to improve the woman’s seizure frequency. An MRI did not demonstrate any structural brain abnormalities. An epilepsy monitoring unit (EMU) evaluation captured seizures with a broad, right hemispheric onset. An initial MEG recording prior to VNS implantation captured epileptiform activity, which was originally and unsuccessfully analyzed using ECD modeling. The interpretation at the time was that the MEG study did not provide localizing information.

**Figure 1:**
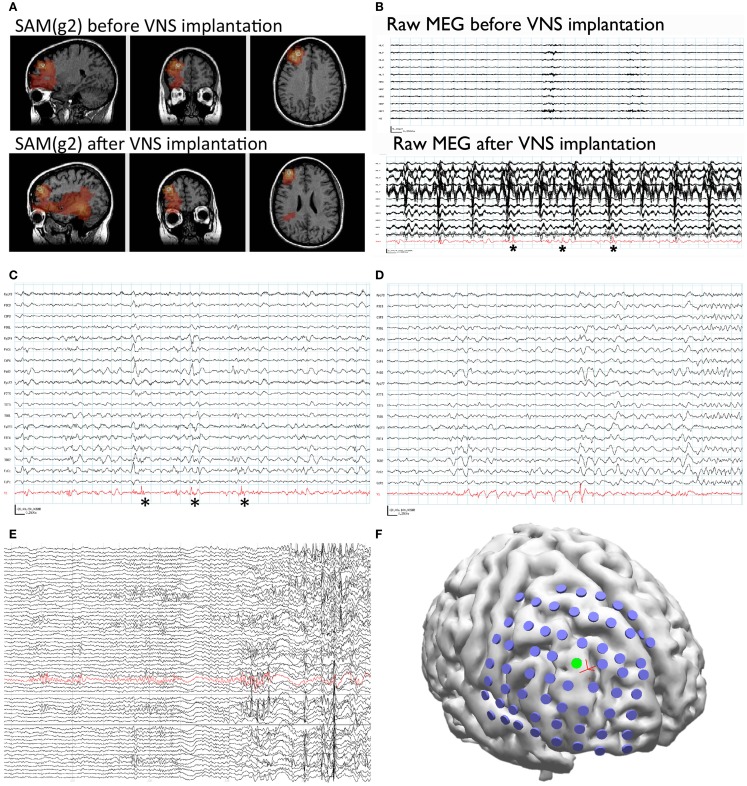
**Patient 1**. **(A)** SAM(g2) analysis of the MEG recording before VNS implantation (upper panel) and after VNS implantation (lower panel) identifies a peak of kurtosis at the same anatomical location. **(B)** The raw MEG recording (black traces, displayed as a butterfly plot) before VNS implantation (upper panel) and after VNS implantation (lower panel). The raw MEG recording after VNS implantation was heavily contaminated by artifact from the patient’s VNS, while the source signal series (lower panel, red trace) permitted the identification of epileptiform activity (lower panel, asterisks). **(C)** Epileptiform activity (asterisks) identified within the source signal series (red trace) coincided with poorly localized activity on the simultaneously recorded scalp EEG (black traces). **(D)** A run of epileptiform activity was seen in the source signal series (red trace) prior to a poorly localized discharge that was observed on scalp EEG (black traces). **(E)** Electrocorticography was used to identify an ictal focus (red trace). **(F)** Reconstruction of the patient’s brain from her own MRI illustrates the placement of the subdural grid (blue disks). The electrode that was determined to overlie the ictal focus [green disk, red trace from **(E)**] co-localized with the peak identified from the MEG recording (red cross).

Synthetic aperture magnetometry (g2) was used to analyze a second MEG recording after the VNS implantation, as well as the previous recording performed prior to VNS implantation. Even though the second recording was separated from the first by 6 years and the raw MEG sensor data were strongly contaminated by artifact from the VNS during the second recording, an equivalent right frontal focus was identified on both recordings (Figure 1A). This indicates that SAM can reproducibly localize interictal epileptiform activity despite the presence of large artifacts due to the VNS implant. Examination of the source signal series reconstructed from the focus demonstrated MEG discharges that correlated with the simultaneously recorded scalp EEG during the second recording (Figure 1C), and, importantly, that lacked the high-amplitude fluctuations present in the raw MEG data that were induced by the VNS (Figure 1B). (For comparison, the raw MEG sensor data prior to VNS implantation are depicted in the top part of Figure 1B.) Furthermore, during the patient’s second MEG recording an electrographic seizure was recorded by EEG, with a preceding MEG-recorded discharge detected in the source signal series (Figure 1D). The SNR for the virtual electrodes was at least 50:1.

Given the new localizing information provided by the MEG recordings, the patient was determined to be a candidate for invasive monitoring and possible resection. A subdural grid was placed over the right frontal lobe, covering the focus identified on MEG (Figure 1F). Seizures captured during the invasive monitoring demonstrated electrographic onset (Figure 1E) very close to the focus of peak kurtosis identified by SAM(g2) on the MEG recordings (Figure 1F). Following resection, which included the focus identified by SAM(g2), the patient experienced a significant improvement in her seizures, improving from four to six focal seizures with loss of awareness and sometimes evolution to bilateral convulsive seizures monthly to two to four focal seizures with retained awareness monthly. Her scalp EEG recordings demonstrated a greatly reduced frequency of interictal epileptiform activity.

#### Patient 2

A 58-year-old man with VNS implantation 2 years prior to his MEG recording presented for further evaluation. The patient’s seizures began at age 1 year old after an episode of meningitis. His seizures began with staring and fidgeting with his hands and exclamations of “Thank you Jesus.” The seizures occasionally evolved to bilateral convulsive seizures. Seizure frequency did not improve with phenobarbital, phenytoin, primidone, carbamazepine, valproic acid, oxcarbazepine, topirimate, levetiracetam, zonisamide, or pregabalin. An MRI of his brain revealed left hippocampus sclerosis. Upon EMU admission, several seizures were captured, with localization to the left frontotemporal region.

**Figure 2:**
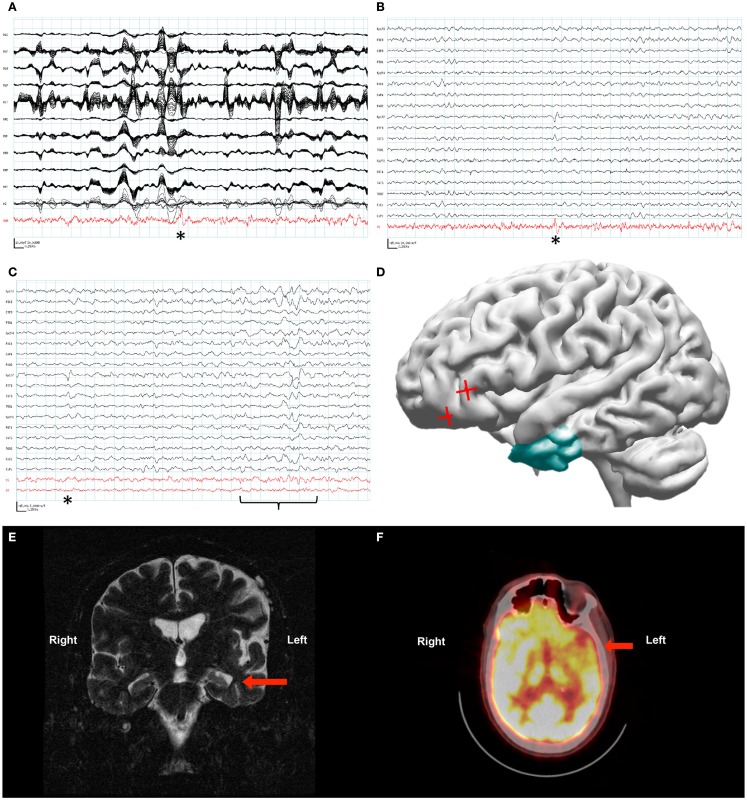
**Patient 2**. **(A)** The raw MEG recording (black traces) was heavily contaminated by artifact from the patient’s VNS, while the source signal series (red trace) allowed identification of epileptiform activity (asterisk). **(B)** Epileptiform activity (asterisk) identified within the source signal series (red trace) coincided with the patient’s frequent interictal spike on the simultaneously recorded scalp EEG (black traces). **(C)** Source signal series at the site of the temporal lobe depth electrodes (red traces, V1 left temporal lobe, V2 right temporal lobe) did not demonstrate a change in activity with the left frontotemporal spike seen on scalp EEG (asterisk). A change in the source signal series from the left temporal lobe correlated with left-sided slowing seen on scalp EEG (bracket). **(D)** Reconstruction of the patient’s brain from his own MRI illustrates the left temporal resection (green) as well as the likely source of his interictal activity based on SAM(g2) analysis of his MEG recording (red crosses). **(E)** A coronal plane T2-weighted MRI of the patient’s brain demonstrating sclerosis of the left hippocampus (arrow). **(F)** An axial plane ^18^F-FDG PET image demonstrating hypometabolism of the left frontotemporal region (arrow).

The MEG recording captured the patient’s most frequent interictal epileptiform activity, a left frontal spike, as seen on the simultaneous scalp EEG. The original data analysis was complicated by the VNS-induced artifact, and the attempted localization was performed by marking spike times in the EEG, and ECDs were then computed from the MEG data on the corresponding times. The initial interpretation of spikes in the MEG recording analyzed with ECD was only that the spikes lateralized to the left hemisphere. However, later analysis of this MEG recording with SAM(g2) identified a peak in kurtosis in the left orbital frontal cortex in spite of the artifact from the patient’s VNS (Figure 2A). The source series reconstruction for this focus lacked the high-amplitude artifact present in the raw MEG sensor data, and epileptiform activity with an SNR of at least 35:1 was evident in the clean source signal series for this voxel, which was coincident with epileptiform activity seen by scalp EEG (Figure 2B).

As the localizing information from the beamformed MEG data was not available initially, placement of invasive monitoring was planned based on the abnormalities seen on MRI (left mesiotemporal sclerosis, Figure 2E) and positron emission tomography (PET) (left frontotemporal hypometabolism, Figure 2F). Three depth electrodes were placed into each temporal lobe. A total of seven strip electrodes were placed over the frontal and temporal cortex bilaterally, although the peak that was later identified from the MEG beamforming was not covered. Several seizures were captured with a possible focus in the left anterior temporal lobe, and the patient had a left anterior temporal lobectomy with amygdalohippocampectomy (Figure 2D). Following resection the patient’s seizure frequency improved slightly, from about four seizures monthly to two to three seizures monthly. His seizure semiology changed in that he no longer verbalized at the beginning of his seizures. However, the patient’s notable interictal epileptiform activity on scalp EEG, a left frontotemporal spike, remained unchanged following his resection. Further analysis of the MEG recording with source signal analysis at the sites of the left temporal depth electrodes and the right temporal depth electrodes identified MEG activity in the left temporal lobe that correlated with episodes of left temporal slowing on the scalp EEG and that appeared independent of the left frontotemporal spikes (Figure 2C).

#### Patient 3

**Figure 3:**
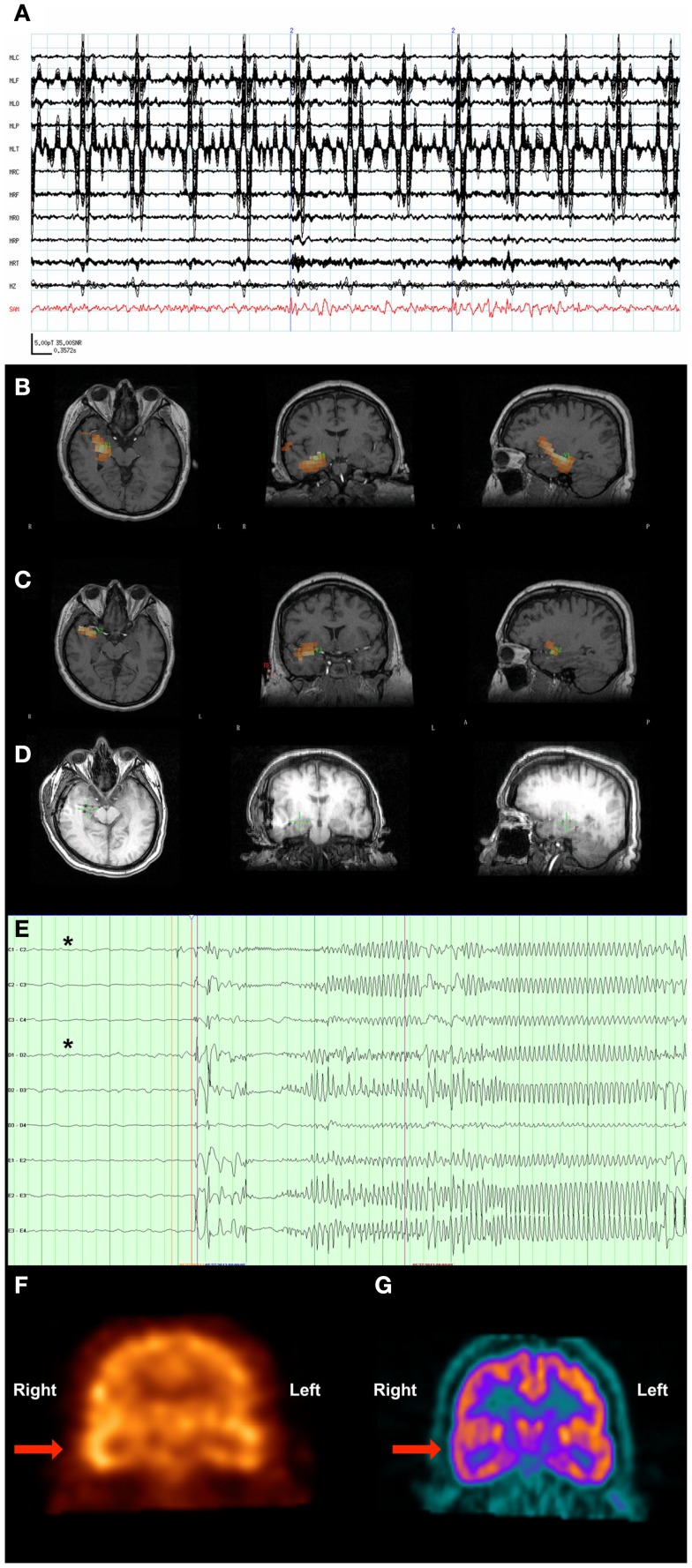
**Patient 3**. **(A)** The raw MEG sensor data (black traces) exhibited strong artifacts due the patient’s VNS, but the source signal reconstruction (red) from the amygdala displayed clear spikes. **(B)** The SAM(g2) statistical parametric maps indicated a right hippocampal focus as well as **(C)** another focus in the amygdala. **(D)** A CT scan reveals the placement of the hippocampal and amydalar depth electrodes, as well as the location of the hippocampal focus (green cross) as identified by SAM(g2). **(E)** An example of a seizure that arose from the anterior hippocampal and amygdalar electrodes, (black asterisks). **(F)** A coronal plane ictal SPECT image demonstrating hyperperfusion of the right temporal lobe (arrow). **(G)** A coronal plane ^18^F-FDG PET image demonstrating hypometabolism of the right temporal lobe (arrow).

A 37-year-old man with a VNS presented for further evaluation. The patient had focal seizures with impaired awareness and occasionally evolution to bilateral convulsive seizures since he was 18 years old. Several events were captured during an EMU admission, all of which localized to the right temporal region. An MRI of his brain did not reveal any structural abnormalities. Single-photon emission computed tomography (SPECT) (Figure 3F) and PET imaging (Figure 3G) suggested that a right temporal seizure focus. An MEG recording analyzed by SAM(g2) revealed a right mesial temporal focus centered on the hippocampus (Figure 3B) and amygdala (Figure 3C), with some occasional right ventral frontal, right posterior, and lateral temporal spread from this zone, and some additional spread to right insula. [While MEG is less sensitive to deep sources, others have successfully imaged hippocampus and amygdala (41–43), and given the invasive monitoring results and resection outcome listed below, the hippocampal and amygdalar sources seem reasonable.] While the raw MEG sensor data exhibited artifacts due the VNS, the source signal reconstructions from hippocampus and amygdala (Figure 3A) displayed no evidence of VNS artifact, with an SNR of 35:1 for the interictal spikes. While clear spikes existed in the source signal data, the EEG data only occasionally exhibited simultaneous interictal spikes.

Invasive monitoring was planned on the basis of the concordant findings between MEG, SPECT, and PET. Subdural grids were placed over the lateral and mesial aspects of the right temporal lobe, and depth electrodes were inserted into the right amygdala and right anterior and posterior hippocampus. Frequent interictal spikes were seen on the hippocampal and amygdalar electrode contacts (Figure 3D), and several seizures arose from the anterior hippocampal and amygdalar electrodes, an example of which can be seen in Figure 3E.

Following the invasive monitoring the patient received a right anterior temporal lobectomy with amygdalohippocampectomy. Prior to his surgery, the patient experienced ~2 seizures per month. Following surgery, the patient was seizure-free for several months, but experienced a breakthrough of two seizures following a reduction in lacosamide dosage, and another breakthrough of four seizures coincident with the onset of an illness.

#### Patient 4

A 63-year-old right-handed woman with VNS implantation 6 years prior to her MEG recording presented for further evaluation. Since age 18 months old the patient had focal seizures with impaired awareness and occasional evolution to bilateral convulsive seizures. Her auras were typified by staring off, ringing in her ears, and a nauseated feeling. The patient experienced feelings of déjà vu prior to bilateral convulsive seizures. Prior EEG evaluation had revealed bitemporal interictal spikes, more frequent over the left than the right hemisphere. Seizures were captured and appeared electrographically generalized. The patient had a choroid cyst adjacent to her right hippocampus, but otherwise the MRI of her brain was unremarkable. Primidone, lacosamide, and lamotrigine had not provided adequate seizure control. An MEG recording, beamformed with SAM(g2), indicated widespread activity with a left lateral temporal as well as left lateral frontal activity and a left mesial occipital–parietal focus. Source signal series from these areas displayed no evidence of VNS artifact and had an SNR of at least 35:1 for the interictal spikes relative to baseline activity.

Based on information obtained from her EEG and MEG recordings, the patient underwent invasive monitoring. Bilateral subdural strips were placed in the orbitofrontal, lateral temporal, and inferior temporal regions. Bilateral depth electrodes were also placed in the amygdale and anterior and posterior hippocampi. Because, a resection of the occipital/parietal focus would have likely resulted in deficits, it was not covered by grids or strips.

The invasive monitoring captured six seizures arising from the right hemisphere and seven seizures arising from the left hemisphere. Of this total, five events were electrographic seizures and two were electro-clinical seizures. The monitoring revealed that the patient had independent seizures arising from broad fields in both hemispheres. On this basis, it was felt that the patient was a poor surgical candidate for seizure reduction or freedom, and the grids and strips were explanted.

#### Patient 5

A 36-year-old right-handed woman with implantation of a VNS 6 years prior to her MEG recording presented for further evaluation. The patient had focal seizures with a sensory aura and impaired awareness following a childhood head injury. Her seizures were pharmaco-resistant, and evaluation with scalp EEG suggested that a left temporal focus. EMU evaluation 1 month prior to her MEG scan captured episodes of generalized spike-wave discharges. An MRI of her brain did not demonstrate any structural abnormalities. The woman’s MEG recording, beamformed with SAM(g2), revealed foci in the left dorsal frontal cortex, bilateral anterior insula, right prefrontal cortex, and left occipital cortex. The VNS artifact was absent from the source signal reconstructions for these areas, and all interictal spikes had an SNR of at least 35:1. The spikes present in the source signal series corresponded with spikes in the accompanying EEG, though the EEG spikes had a broad, left hemispheric field.

#### Patient 6

A 6-year-old girl with a VNS implantation for pharmaco-resistant seizures presented from an outside institution for further evaluation. The patient was known to have a cortical dysplasia. The patient’s EEG revealed numerous distributed spikes, suggesting that the presence of multiple foci. Beamforming of the MEG recording with SAM(g2) revealed bilateral orbitofrontal activity with a much more kurtotic signal on the left than the right. SAM(g2) also identified bilateral temporal activation with a much greater involvement of the right hemisphere. There was an additional focus of lesser kurtosis encompassing the left insula and spreading to the left occipitoparietal cortex. The source signal reconstructions corresponding to these foci possessed an SNR of about 35:1 and exhibited numerous interictal spikes with simultaneous EEG spikes.

#### Patient 7

A 40-year-old right-handed man with VNS implantation 7 years prior to the MEG recording presented for further evaluation. He had received a right anterior temporal lobectomy 2 years prior to MEG recording. Since age 2 years old the patient has had focal seizures with an autonomic aura and evolution to bilateral convulsive seizures. Multiple seizures were captured during EMU monitoring and which could only be lateralized to the right hemisphere. An MRI of his brain demonstrated only his prior resection, and SPECT did not identify a seizure focus. SAM(g2) analysis of his MEG recording revealed a right posterior lateral parietal focus just dorsal to the lateral fissure whose spatial extent included posterior parts of the superior and middle temporal gyri. This activity was located slightly above the resection margin. The SNR for this source signal series was >20:1, although the interictal spikes present in the source signal series did not have a clear EEG correlate during the simultaneous recording.

#### Patient 8

A 47-year-old woman with VNS implantation 10 years prior to her MEG recording presented for further evaluation. The patient reported focal seizures with evolution to bilateral convulsive seizures since childhood. The patient’s baseline EEG was notable for bilateral, independent temporal spikes. Despite multiple admissions for EMU monitoring and several captured seizures, scalp EEG demonstrated only subtle changes that were neither localizing nor lateralizing. The patient’s MRI demonstrated right mesial temporal sclerosis, bilateral polymicrogyria located around the lateral fissures, and a left hemisphere closed-lip schizencephaly. Analysis of her MEG data by SAM(g2) revealed kurtotic activity that localized to left lateral parietal cortex just above the lateral fissure and extended down into the superior temporal gyrus. Importantly, this focus overlaps with the region of schizencephaly in the left parietal cortex. The source signal series for this focus had an SNR of about 35:1, although the interictal spikes present in the MEG recording did not have a clear EEG correlate.

Given the findings of right mesial temporal sclerosis and the probable multifocal nature of this patient’s epilepsy, this patient received a right temporal lobectomy without invasive monitoring with the goal of seizure reduction. Prior to her surgery, the patient had two to eight focal seizures with evolution to bilateral convulsive seizures monthly, and following resection no seizures have been reported although the patient continues to have likely non-epileptic spells.

## Discussion

Synthetic aperture magnetometry (g2) is a beamformer analysis method that can transform biomagnetic time series into functional images and also allows the derivation of source signal series for those locations. The presented cases demonstrate an expanded use of clinical MEG to patients with VNS through the application of the SAM(g2) beamformer. Patients with medically refractory epilepsy often are treated using devices such as VNS, and may need more exhaustive surgical evaluations including MEG. Despite severe artifacts that obscure meaningful interpretation of the raw MEG data, here the analysis by SAM(g2) identified focal areas of high kurtosis that were found to be concordant with additional structural, electroencephalographic, or metabolic studies. These cases indicate that a VNS is not a contraindication for MEG and that valid and clinically meaningful results can be obtained through SAM(g2) analysis of MEG recordings from patients with a VNS.

In several of the cases presented, the MEG and SAM(g2) analysis provided results that further informed surgical decisions. Several studies have retrospectively or prospectively studied the role of MEG in surgical planning (20, 33, 44–51). The American Clinical MEG Society (ACMEGS) has also issued a position statement supporting the value of MEG in pre-surgical evaluation (52). Our case series adds to this body of data by describing the use of the SAM(g2) beamformer to perform magnetic source imaging for patients with a VNS implant.

Several lines of evidence from these patients indicate the feasibility of using SAM(g2) to identify foci from MEG recordings in individuals with a VNS. (1) Patient 1 received MEG scans before and after VNS implantation, and the focus for both scans was identical. This indicates that localization by SAM(g2) is not altered by the VNS artifact. (2) Patients 1, 3, and 4 had invasive electrographic monitoring of the foci identified by beamforming, and the results of the monitoring were consistent with the MEG findings. Additionally, patients 5 and 6 had multifocal epileptiform activity that was consistent on both their MEG recordings and electrographic monitoring. (3) Patients 7 and 8 had structural brain abnormalities, and MEG foci co-localized with these abnormalities. (4) None of the patients had a MEG finding that contradicted observations made with EEG or structural or metabolic imaging.

Localization of seizure foci in cases of pharmaco-resistant epilepsy remains a difficult problem. With increasing emphasis on treating pharmaco-resistant epilepsy with surgical resection (53, 54), additional methods are needed to accurately and efficiently identify appropriate surgical candidates. Additionally, non-invasive methods are needed to identify the brain regions to be monitored with spatially restricted invasive techniques (e.g., patients 1–3), or to identify individuals as unlikely to benefit from invasive monitoring or surgery due to the number or location of seizure foci (e.g., patients 5 and 7).

Magnetoencephalography captures the activity of the whole brain and can accurately localize activity to an individual’s own MRI. When MEG data are analyzed with SAM(g2), this powerful combination appears to fulfill important requirements of greater accuracy, reliability, and robustness. Using SAM(g2), foci could be localized that could not be previously analyzed and determined by visual inspection or with traditional ECD analysis. Reliable results were obtained when comparing VNS and non-VNS implant conditions, even when the MEG recordings were 6 years apart. We conclude that SAM(g2) provides unique information that is diagnostically useful, and that with appropriate analysis MEG should not be excluded from the evaluation of individuals with a VNS.

## Conflict of Interest Statement

The authors declare that the research was conducted in the absence of any commercial or financial relationships that could be construed as a potential conflict of interest.
